# Usability and Safety of the ATLAS 2030 Robotic Gait Device in Children with Cerebral Palsy and Spinal Muscular Atrophy

**DOI:** 10.3390/children11121500

**Published:** 2024-12-10

**Authors:** Carlos Cumplido-Trasmonte, Eva Barquín-Santos, Fernando Aneiros-Tarancón, Alberto Plaza-Flores, Sandra Espinosa-García, Roemi Fernández, Elena García-Armada

**Affiliations:** 1 Marsi Bionics S.L., 28521 Madrid, Spain; carlos.cumplido@marsibionics.com (C.C.-T.); eva.barquin@marsibionics.com (E.B.-S.); fernando.aneiros@marsibionics.com (F.A.-T.); alberto.plaza@marsibionics.com (A.P.-F.); elena.garcia@marsibionics.com (E.G.-A.); 2 International Doctoral School, Rey Juan Carlos University, 28922 Madrid, Spain; 3 Physical Medicine and Rehabilitation Service, La Paz University Hospital, 28046 Madrid, Spain; sandra.espinosa@salud.madrid.org; 4 Centre for Automation and Robotics CAR CSIC-UPM, Spanish National Research Council, Ctra. Campo Real Km 0,200 La Poveda, Arganda del Rey, 28500 Madrid, Spain

**Keywords:** cerebral palsy, spinal muscular atrophy, robotics, rehabilitation, gait, children

## Abstract

Purpose: the purpose of this study was to evaluate the safety and usability of the ATLAS 2030 in children with Cerebral Palsy (CP) and Spinal Muscular Atrophy (SMA). Materials and Methods: the sample consisted of six children, three with CP and three with SMA, who received eight sessions of robot-assisted gait therapy. Safety was measured by the presence of adverse events. Usability was measured by spatiotemporal parameters, the Six-Minute Walking Test (6MWT), and the time needed for donning and doffing, as well as satisfaction questionnaires administered to therapists and patients. Results: no serious adverse events were reported. The average cadence and number of steps per session increased throughout sessions, as well as the distance covered in the 6MWT, both in participants with CP and SMA. The mean donning time at the end of the study was 4.6 ± 1.3 min, and only one therapist was necessary to carry out all of the sessions. Satisfaction was considered high by both children and therapists. Conclusions: the ATLAS 2030 was shown to be safe for children with CP and SMA. The usability of the device was good, since a progression in the spatiotemporal parameters was observed throughout the sessions, and patient and therapist satisfaction were high.

## 1. Introduction

According to the World Health Organization’s World Report on Disability, approximately 93 million children worldwide have some form of moderate disability, and 13 million have severe disabilities. These conditions often lead to secondary issues that impact quality of life, life expectancy, and daily functioning and participation [1]. Among the conditions that can result in childhood disability, Cerebral Palsy (CP) and Spinal Muscular Atrophy (SMA) are frequently associated with impaired walking ability [2,3]. CP is the most common motor disability in childhood, with an incidence of 2–2.5 per 1000 births [4]. It was defined by Rossenbaum as “a group of permanent disorders of the development of movement and posture, causing activity limitation, that are attributed to non-progressive disturbances that occurred in the developing foetal or infant brain” [5]. One of the most important problems related to this disease is motor disability, as 28% of the children with CP cannot walk, and 16% need an assistive device [6].

SMA is a congenital neuromuscular disease [7], with an incidence of 1–2 per 100,000 people in the world, and it is considered the most common genetic cause of infant mortality [8]. It is caused by the mutation of the SMN-1 gene that makes motor neurons in the spinal cord degenerate, leading to generalized muscle weakness and atrophy [3,7]. Since it is a progressive and degenerative disease, dependence for the activities of daily living (ADLs) increases over the years, as well as the health problems related to muscle weakness and gait loss [9].

Conventional therapy, such as the use of orthoses to minimize joint contractures, muscle strength training, and gait training, is recommended, when possible, in both SMA and CP [10,11,12]. Rehabilitation goals can be met by different therapy approaches, such as goal-oriented exercises, intensive training, and performing ADLs [9,13]. Evidence supports the effectiveness of motor interventions that have the following characteristics in common: (a) they are based on the practice of specific tasks and ADLs; (b) self-initiated or self-generated movements are used; (c) they are performed intensively; and (d) they are goal-oriented and measurable [13]. Robot-assisted gait training complies with all of these characteristics [14,15,16]. Walking constitutes a standing work, strengthens the musculature of the lower limbs and trunk, and exercises the lower limbs to maintain mobility. Robotics has the advantage of providing repetitive and intensive task-oriented therapy (such as walking), which enhances neuroplasticity and motor learning [17].

In recent years, there has been a significant increase in the number of studies published on pediatric robotic gait devices [18]. The evidence regarding robot-assisted gait training in children with CP is still scarce, although it has shown promising results [19,20,21]. Regarding SMA, evidence is even more limited, and the only studies published so far used the overground robotic device ATLAS 2030, suggesting potential improvements [22,23,24]. However, the safety and usability of this device have not yet been tested in these populations. Therefore, the aims of the present study are (1) to analyze the safety of the ATLAS 2030 in a sample of children with CP and SMA based on the occurrence of adverse events and significant changes in vital signs; and (2) to investigate the usability of the ATLAS 2030 by assessing spatiotemporal parameters, device malfunctions, fatigue, and performance in the 6MWT while wearing the device, as well as the degree of satisfaction reported by patients and therapists.

## 2. Materials and Methods

### 2.1. Participants

Participants were recruited by a pediatric physician from La Paz University Hospital (Madrid, Spain). All of them were assessed to ensure they met the following inclusion criteria: age between 3–14 years; weight lower than 35 kg; femoral length (from hip joint to knee joint in the sagittal plane) between 22–38 cm; tibial length (from knee joint to ankle joint in the sagittal plane) between 21–37 cm; distance between greater trochanters between 24–40 cm; confirmed diagnosis of CP or SMA; stable medical condition without changes in disease-modifying medication in the last 6 months; patient under medical follow-up according to the normal standards recommended for their disease; no need for daytime mechanical ventilation (normal oxygen saturation and PCO2 with ambient oxygen); and the inability to walk without assistance (defined as the inability to walk 10 m without help, support, or assistance). Exclusion criteria were the inability to understand simple commands, to report basic needs, or to actively collaborate in the therapy; scoliosis with a Cobb angle higher than 25 degrees, without the possibility of wearing a brace during the test; severe skin alteration on the lower extremities; and surgical intervention scheduled within the next 6 months, or performed within the last 6 months.

### 2.2. Device

**Figure 1:**
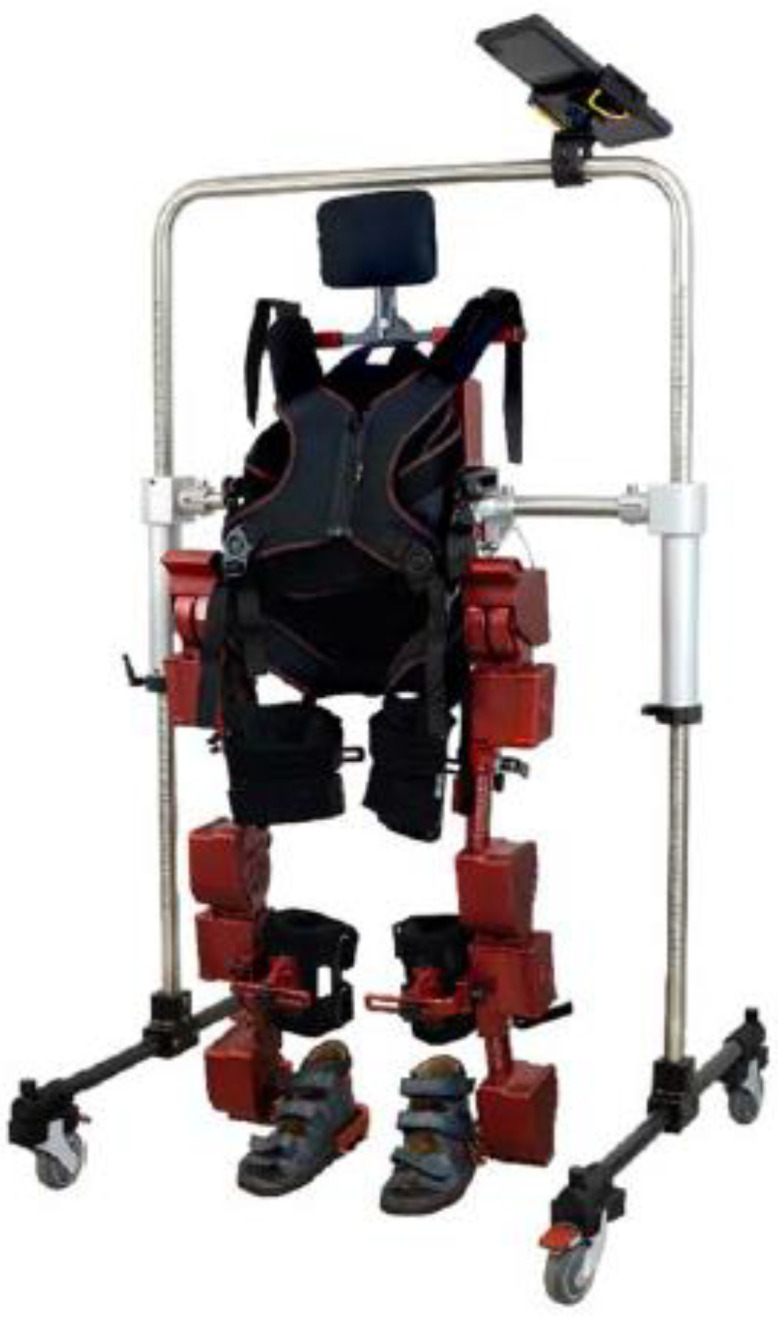
ATLAS 2030 robotic device.

ATLAS 2030 (Marsi Bionics S.L., Madrid, Spain) is an overground pediatric robotic device for gait training [22] (Figure 1). Specifically, it is the only commercially available fully portable pediatric robotic gait device, since other similar devices on the market are fixed, use a treadmill, or need external adaptations such as weight support devices [18,25,26]. This device is CE-cleared for use in children with CP and SMA. These two conditions provoke different motor symptoms, since CP usually causes spasticity [5,27], whereas SMA courses with hypotonia [3]. However, they result in similar levels of disability, since they both lead to restrictions of the range of motion, weakness, and an impaired walking capacity, affecting social interaction and quality of life.

The ATLAS 2030 is attached to the body in a non-invasive way and must be used and controlled by a certified clinician. It has eight motors that allow for hip, knee, and ankle flexion and extension, and hip abduction; additionally, its size can be adapted depending on leg length and hip width. Its ergonomics ensure an optimum body alignment and allow children with a lack of trunk control to stand and walk, achieving a physiological gait pattern. The device allows children to perform sit-to-stand exercises, staying in a standing position, as well as walking forwards and backwards. It operates in the following two different modes, depending on the level of assistance required: (1) automatic mode, where the device generates all of the force necessary to move the child’s legs for stepping; and (2) the active-assisted mode, where the patient must initiate the step, since the device detects the intention of movement and assists in providing the assistance needed to accomplish the step. The ATLAS 2030 is always used with a safety frame that ensures stability while standing, sitting, or walking, and allows clinicians to work in front of the child to perform rehabilitative exercises whilst walking.

### 2.3. Study Design

This study is a prospective case series and was conducted at the Marsi Care research facilities, at the Centre for Automation and Robotics from the Spanish National Research Council and the Technical University of Madrid. The study was approved by the Comunidad de Madrid Regional Research Ethics Committee with Medical Products (reference 47/370329.9/19), and all parents or legal guardians of the participants received and signed the informed consent form, according to the Helsinki Declaration [28]. The clinical trial was registered at Clinical Trials.gov: NCT04837157 on 4 August 2021.

The trial consisted of 11 appointments, divided into an initial telephone screening call (visit 0); a screening visit (visit 1); 8 sessions with the ATLAS 2030 twice per week (from visit 2 to 9); and a follow-up 72 h after visit 9 (visit 10). All visits were carried out by just one physiotherapist who was fully trained in the use of the device.

During visit 1, inclusion and exclusion criteria were assessed for each participant. In addition, anthropometric measurements were taken to ensure the proper adjustment of the device, followed by a trial with the device, and the performance of the 6MWT while wearing it. The 8 gait training sessions with the ATLAS 2030 (from visit 2 to visit 9) lasted a maximum of 60 min, and all of the participants performed the same exercise protocol, as follows: (1) sit-to-stand transfers (10 min); (2) maintaining standing position (10 min); (3) forward and backward walking in automatic and active-assisted modes (10 min in each mode); (4) trunk rotation exercises while walking in automatic mode (10 min); (5) balloon or ball games while walking in automatic mode (5 min); and (6) balance exercises in standing (5 min). The 6MWT was tested again at visits 5 and 9 to assess the progression of the patient’s performance.

### 2.4. Patient-Related Outcome Measures

The evaluation procedure is shown in Table 1.

#### 2.4.1. Safety

Safety was assessed by the occurrence of adverse events (AEs), such as falls, while walking with the device; a pain increase in 3 points measured by the Wong–Baker FACES^®^ Pain Scale [29] after using the device; any physical injury caused by the device; or any serious adverse event (SAE), as defined by the United States of America Food and Drugs Administration (FDA) (event that causes death, hospitalization, disability, congenital anomaly, is life-threatening, or that requires intervention to prevent permanent impairment or damage) [30].

Vital signs were measured before and after each session. Heart rate (beats per minute), systolic and diastolic blood pressure (mmHg), and oxygen saturation (% SpO_2_) were measured using the PC-900PRO^®^ vital signs monitor (Creative Medical^®^, Shenzhen, China). Respiratory rate was taken manually by counting the number of breaths (by observing the movement of the chest as the child breathed) in one minute using a stopwatch.

#### 2.4.2. Usability

Usability was assessed by measuring the progression of spatiotemporal parameters (walking time and total number of steps taken per session) with the device, and the degree of fatigue after walking with the device using the Children’s OMNI Perceived Exertion Scale [31]. Furthermore, the participants performed the 6MWT while using the device in the active-assisted mode at visits 1, 5, and 9 to assess performance progression. In addition, any device malfunction was recorded.

To assess satisfaction, a Spanish version of the Quebec User Evaluation of Satisfaction with Assistive Technology (QUEST) 2.1: Children’s Version [32] was administered to the children, which can be filled either by the children, the parents/caregivers, or both.

### 2.5. Therapist-Related Outcome Measures

#### 2.5.1. Safety

Safety was assessed by the recording the occurrence of adverse events (AEs), such as falls, while using the device; a pain increase in 3 points measured by the Visual Analogue Scale (VAS) after using the device; any physical injury caused by the use of the device; or any serious adverse event (SAEs), as defined by the United States of America Food and Drugs Administration (FDA) (event that causes death, hospitalization, disability, congenital anomaly, is life-threatening, or that requires intervention to prevent permanent impairment or damage).

#### 2.5.2. Usability

Usability was evaluated by the degree of fatigue reported by the therapist after using the device, measured by the Borg scale [33], as well as by collecting the level of assistance (number of therapists) required to transfer patients to the device and for donning and doffing, as well as donning and doffing time. Therapist satisfaction was assessed using the QUEST 2.0 (adult version) at the end of the study [34]. The questionnaire consists of 12 satisfaction items, with a final section where the interviewee selects the 3 items considered to be the most important [35]. In addition, the System Usability Scale (SUS) [36] was administrated to the therapists to assess the overall device usability.

### 2.6. Statistical Analysis

The analyses were performed by classifying subjects by disease. As the sample consisted of three participants with CP and three with SMA, a descriptive analysis of all quantitative data was carried out. Data were synthesized and statistically analyzed, as appropriate, through frequencies of occurrence, means, and standard deviations. The scores of the questionnaires are expressed in median and interquartile range, as they were qualitative questionnaires. The results were visualized through line graphs and contingency tables. All calculations were performed with IBM SPSS Statistics v27 software (IBM Corp., New York, NY, USA) and Microsoft Excel 2019 (Microsoft Corp., Washington, DC, USA).

## 3. Results

### 3.1. Participants-Related Results

Seven children were recruited through an initial phone call (V0) by the rehabilitation service of La Paz University Hospital (Madrid, Spain). However, one of them was excluded at the initial screening (V1) because their femur exceeded the maximal upper leg length. The mean age was 6.6 ± 1.9 years, the mean weight was 20.9 ± 3.9 kg, and the mean height was 112.2 ± 5.5 cm. All subjects with SMA were under treatment, with Spinraza^®^ administered every 4 months from the age of two years (P2 and P6) and three years (P1). Table 2 details the individual sociodemographic data for each subject.

There were no falls, physical injuries, or SAE related to participants during the study, and neither were there episodes of pain that interfered with the sessions. However, there were five minor pain episodes, four of them categorized as level 1 (out of 10), according to the Wong–Baker scale, and one categorized as level 2. Three of these pain episodes affected children with SMA and two affected a participant with CP. The pain episode categorized as level 2 was due to foot discomfort and was relieved shortly after sitting. None of these episodes entailed an increase in pain of 3 points. Every episode of pain happened from visits 1 to 3, except for the episode scored as level 2, which happened at visit 7.

Data gathered related to vital signs before and after the use of the device is shown in Table 3. No significant change was found in vital signs after the use of the device.

Concerning device malfunctions, only one failure was recorded during the study, and it consisted in an excessive right hip flexion performed in slow motion. The session was stopped, and the therapist explored the participant and the device. Since no harm or damage was detected, both the participant and device were able to continue the trial. This event did not affect data collection.

**Figure 2:**
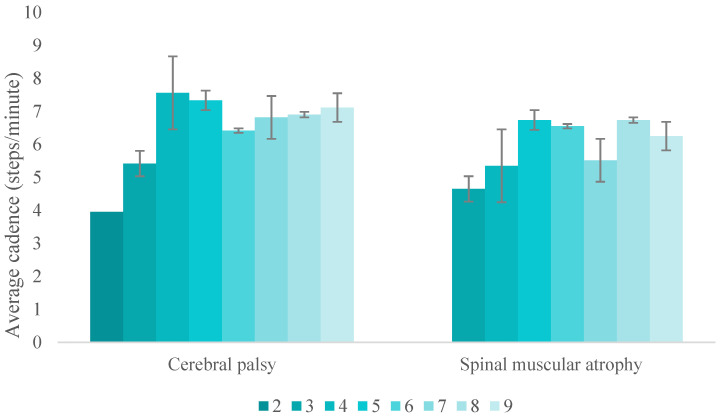
Average cadence per visit (2–9) and pathology. Note: In the second session, no data are reflected in the participants with SMA. This was since, in that session, they did not walk due to fear of walking. Error bars show one standard deviation.

The overall usage time of the device was 44.3 h divided into 52 sessions. Usage time per visit (excluding visit 1, given the adaptation process needed for this first session) ranged from 39.6 min to 60 min, with a mean of 57.3 ± 7.0 min for CP and 53.5 ± 12.0 min for SMA. The overall number of steps was 5454, and the average number of steps per visit for participants with CP was 120.8 ± 32.7 and 106.4 ± 43.2 for participants with SMA. At visit 1, where participants used the device for the first time, an average of 80.5 steps were taken. Nevertheless, at the last session (V9), participants walked an average of 133.67 steps. On average, participants walked 909.0 ± 94.2 steps across the study, with a range from 786 to 1023 steps. The average cadence was 6.3 ± 1.4 steps per minute. Cadence progression across sessions classified by disease can be observed in Figure 2.

The mean degree of fatigue measured by the OMNI scale was 1.5 ± 1.8 points for children with CP and 1.8 ± 1.7 points for children with SMA. Regarding the degree of fatigue reported by therapists, in 75% of the sessions (*n* = 36) there was no fatigue according to the Borg scale. In the remaining 25%, there were slight increases in fatigue, never above 3 points, on the Borg scale.

**Figure 3:**
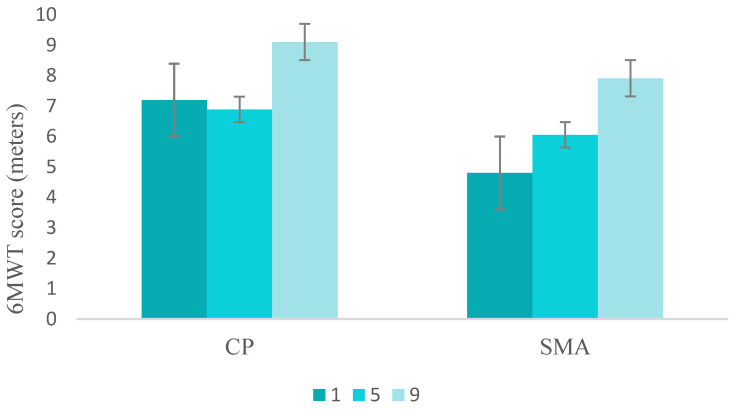
Average meters walked in the 6MWT by pathology and visits (1, 5, and 9). Error bars show one standard deviation. CP: Cerebral Palsy; SMA: Spinal Muscular Atrophy.

Overall, children with CP increased the distance walked in the 6MWT by 2.0 m (up to 24.6%) between the initial and final visit, while children with SMA increased it by 3.1 m (up to 46.4%). The first time that they performed the 6MWT (V1), children with CP increased their heart rate by 12.1%, from 90.6 ± 10.7 to 101.7 ± 5.7 beats/minute. This difference was reduced to 6.0% at the end of the study (V9), from 99.7 ± 12.5 to 105.0 ± 15.7 beats/minute. Children with SMA increased their heart rate by 3.6%, from 112 to 116 beats per minute during 6MWT at V1, and this difference remained constant at the end of the study (3.7%), from 104.3 ± 1.1 to 108.7 to 1.1 beats per minute. It can be observed in Figure 3.

The QUEST 2.1 was answered by the participants with help from the parent/caregiver, except for one case, where it was filled by the parent/caregiver. All results, listed by disease, are shown in Table 4.

From patients’ perception, the most important items were the following, in order of importance: “satisfaction” (32%), “easiness of use” (26%), “reliability” (11%), “easiness to mobilise the device” (11%), “size” (11%), “assistance in malfunction” (5%), and “time it took to get” (5%).

### 3.2. Therapist-Related Results

There were no falls, physical injuries, pain episodes, or SAEs related to therapists during the study.

The average donning time was 6.4 ± 4.2 min, and the average doffing time was 2.6 ± 1.3 min. At the last visit (V9), the average donning time was 4.6 ± 1.3 min and 1.6 ± 0.5 for doffing. Regarding assistance, only one therapist was needed for transferring the child, donning and doffing the device, and conducting the whole session in every visit.

Regarding the results of the QUEST 2.0, all responses are shown in Table 5. Regarding the most important satisfaction items, both therapists agreed that “effectivity”, “easiness of use”, and “safety” were the most important.

The results of the SUS scale regarding usability were 82.5 for one therapist and 77.5 for the other. Therefore, the usability of the device was rated with a mean of 80.0 ± 3.5. Five items had a mean score > 4.5 out of 5. The items with the highest scores were the following: ‘I think that I would like to use this system frequently’ (5.0 ± 0.0); ‘The system was easy to use’ (4.5 ± 0.7); ‘I found the various functions in this system well integrated’ (4.5 ± 0.7); ‘I would imagine that most people would learn to use this system very quickly’ (4.5 ± 0.7); and ‘I felt very confident using the system’ (4.5 ± 0.7).

## 4. Discussion

The present study focuses on analyzing the safety and usability of the ATLAS 2030 in a sample of children with CP and SMA. Patient and therapist safety while using the device was studied, along with other usability-related variables, such as spatiotemporal parameters; assistance required for transferring, donning, and doffing, as well as donning and doffing times; distance walked with the device in the 6MWT; and patient and therapist satisfaction.

Concerning safety, most of the previous research showed similar results to ours, not having reported any SAEs [26,36,37,38,39,40,41]. Nevertheless, a few studies carried out with an overground robotic gait device (HAL Suit^®^, Cyberdine, Japan) in children with CP reported mild adverse events, such as skin peeling and toenail groove breaking [40,42,43]. In addition, some studies with stationary robotic devices have reported SAEs in adults, such as bone fractures [44,45,46,47]. Bessler et al. [48] found that AEs affect 8–13% of the subjects, with soft tissue and musculoskeletal AEs being the most frequent.

Regarding pain, our results agree with those from previous studies with similar devices, were only a few episodes of pain were reported, and they were all considered mild and acceptable [45,46]. However, moderate pain episodes due to the use of these devices have been described in the literature, mainly in stationary robotic devices [47,48,49].

Our results show a trend in AEs related to the use of the device, where 80% of the pain episodes occurred at the beginning of the trial, from visits 1 to 3. Delgado et al. [50] observed a similar tendency when using a portable robotic device in people with spinal cord injury, obtaining results that showed a decrease in AEs with device usage. This tendency could be due to an adaptation of the participant to the device.

The aim of measuring vital signs before and after the use of the device was to detect any significant change in the range of values considered normal for this population. No significant change was found in vital signs after the use of the device, since all parameters remained within normal ranges [51,52,53,54]. However, it is important to note that a very significant or unfavorable change in vital signs could indicate poor usability and safety of the device. The metrics were monitored to ensure the device did not negatively affect physiological stability, and all results fell within normal pediatric ranges. Previous studies in adults with neurological impairment have detected an increase in vital signs after the use of a robotic device, mainly in heart rate and blood pressure, together with a slight perception of effort, although these values were always within the normal range [48,49,55,56].

Only one device malfunction was detected during the trial. This failure forced the session to stop, but it did not cause any harm to the participant, nor the therapist, and both the device and the participant were able to continue the study. To prevent this malfunction from happening again, some improvements were implemented into the device. The number of steps, cadence, and the distance walked in the 6MWT increased in every session, possibly due to the participants’ adaptation to the device. Several studies [57,58,59] have shown significant improvements in the spatiotemporal parameters in children with CP with GMFCS levels III and IV, presenting a more severe motor impairment than the children included in this study. A possible explanation for this would be that these children started with lower baseline spatiotemporal measurements, which may provide a greater capacity for improvement when using robotic assistance. In addition, van Hedel et al. [59] suggest that children with greater impairment, even with a lower walking ability, obtain greater improvements compared to children with lower GMFCS levels. This could also be pertinent to children with SMA, who may benefit the most from robot-assisted gait training, as their walking ability is very limited or even null.

The children included in the study had never had the ability to walk independently. In fact, two children were afraid to stand with the device for the first time, but this fear was overcome after the first session. However, there was an increase in this distance throughout sessions, which suggests a progression in walking speed. Future research should be conducted to support these findings, analyzing the long-term benefits of robot-assisted gait training in the walking capacity of these children.

To the best our knowledge, there are no previous studies that assess the satisfaction of children and/or their parents with robotic devices. Therefore, this study is the first to evaluate user satisfaction with robotic gait devices in children. Participant satisfaction was not as good as the satisfaction reported by the therapists, although some items were scored as “delighted”, specifically the following: “size”, “services received”, and “advice on how to use the device”. On one hand, since the device was not in the market at the time the study took place, they felt less satisfied with the time they had to wait to have access to the technology. On the other hand, it is also possible that these results were associated with the high expectations created by robotic technology in families and children. Many users hope that this technology will fully compensate for their physical limitations and allow them to participate and integrate into society without limitations, and reality collides with these expectations. In the present study, some requests of children and families that could not be met were related to being able to run or turn with the device.

The ATLAS 2030 robotic gait device is currently considered a medical device and, therefore, its use is limited to certified healthcare professionals. Because of that, the device can be adapted to different anthropometric characteristics and complexities, including child growth, as the device is adaptable to different leg lengths and hip widths, with a recommended age of use of 3–14 years (covering much of children’s motor development). Since the ATLAS 2030, to the best of our knowledge, is the only pediatric overground robotic gait device for rehabilitation in the market, this study could be considered as a starting point for its evolution into an assistive device for personal mobility. Donning and doffing times are key factors when choosing rehabilitative technology, since time per session is usually limited in daily clinical practice. In our study, therapists gave the item “device adjustments” 4.5 points out of 5 on the QUEST 2.0, which suggests that the donning and doffing time for the ATLAS 2030 could meet the needs of daily clinical practice. In addition, there is a decrease in the time needed for donning and doffing throughout the trial. While donning took almost 10 min on average at V1 (therapists were not used to the donning process, as it was their first time using the device), this time was reduced to 4 min at V9. The doffing time was reduced from 4 min at V1 to 1.6 min at V9. In order to analyze at which point donning and doffing times settle and stop decreasing, it would be necessary to conduct a larger number of sessions. Previous research with robotic gait devices for adults, such as Indego^®^ (Parker Hannifin Corp., Cleveland, OH, USA), shows similar results when analyzing these variables, with an average donning time of 9:01 min and a doffing time of 2:44 min at the last session of the trial [60,61,62]. Other studies have measured the time needed to reconfigure the device between different patients, obtaining values of 5 min for the Ekso^®^ (Ekso Bionics, San Rafael, CA, USA) and from 5 to 10 min for the Rewalk^®^ (Rewalk Robotics, Inc., Marlborough, MA, USA). However, this configuration can take up to 30 min if the pelvic band and the footplates also need to be adjusted [60]. In addition to adequate donning and doffing times, the fact that only one therapist is needed for donning and doffing the ATLAS 2030, as well as for conducting the therapy, supports the usability of the device.

The QUEST 2.0 is one of the most frequently used questionnaires for assessing the user satisfaction with a robotic device in adults, and it is validated in healthy subjects as well as people with neurological disease [63]. When compared to previous studies that use the QUEST 2.0 to assess therapist satisfaction with other robotic devices, our results are either similar or better [64,65,66]. The degree of satisfaction reported by the therapists that participated in the present study suggests that the ATLAS 2030 is a useful tool from the professionals’ perspective. Regarding the SUS scale, the usability of a device is considered above average if the result is higher than 68, and is considered “good” if the score obtained is from 71 to 85 [36]. The ATLAS 2030 obtained a score of 80 out of 100, which means that it was considered above average and “good” in terms of usability according to the SUS scale [36]. Results in previous similar studies with robotic gait devices for adults showed similar or worse results when assessing usability with SUS [58,67]. Therefore, considering all of the factors mentioned above, the usability of the ATLAS 2030 can be considered adequate.

This study presents certain limitations. First, the sample size was limited. While SMA type II has a low incidence rate, this is not the case for CP. Consequently, the results obtained in this study should be taken with caution before extrapolating them to other children with these conditions. Additionally, the number of sessions can be considered low, potentially obscuring some of the device’s benefits. Future research should prioritize randomized controlled trials (RCTs) with larger sample sizes to confirm these findings. Long-term studies evaluating safety, usability, and efficacy are also necessary to provide a more comprehensive understanding of the impact of the ATLAS 2030 on these populations.

## 5. Conclusions

The ATLAS 2030 seems to be safe and has adequate usability in children diagnosed with CP and SMA type II. Only one therapist was needed for donning and doffing and carrying out the sessions. Additionally, an increase in spatiotemporal parameters, such as the number of steps, cadence, or the distance walked in the 6MWT, has been observed throughout the trial. Furthermore, children and therapists’ satisfaction with the ATLAS 2030 was adequate, showing a good acceptance by both. These results suggest that further research with a larger sample size should be conducted in the future.

## Figures and Tables

**Table 1:** Outcome measures by visit. SAEs: serious adverse events; HR: heart rate; BP: blood pressure; %O_2_: oxygen saturation; RR: respiratory rate; 6MWT: Six-Minute Walking Test; OMNI: Children’s OMNI Perceived Exertion Scale; QUEST: Quebec User Evaluation of Satisfaction with Assistive Technology; SUS: System Usability Scale. Blue: time of the evaluation was carried out.

			V1	V2	V3	V4	V5	V6	V7	V8	V9	V10
PATIENT	SAFETY	Adverse events (Falls, pain, SAEs)										
		Vital signs (HR, BP, %O_2_, RR)										
	USABILITY	Spatiotemporal parameters (Walking time, number of steps)										
		Fatigue (OMNI scale)										
		6MWT (Walking distance in 6 min)										
		Usability Satisfaction (QUEST 2.1)										
THERAPIST	SAFETY	Adverse events (Falls, pain, SAEs)										
	USABILITY	Transfers, don and doff (Time and people needed)										
		Fatigue (Borg scale)										
		6MWT (Walking distance in 6 min)										
		Usability Satisfaction (QUEST 2.0, SUS)										

**Table 2:** Participants’ characteristics. CP: Cerebral Palsy, SMA: Spinal Muscular Atrophy; GMFCS: Gross Motor Function Classification System.

Participant	Sex	Age (Years)	Weight (kg)	Height (cm)	Diagnosis	Classification
1	Male	5	22.3	104	SMA	Type II
2	Male	6	17.9	113	SMA	Type II
3	Male	10	24.0	116	Spastic CP	GMFCS IV
4	Female	8	20.0	120	Dystonic CP	GMFCS IV
5	Female	6	15.5	110	Spastic CP	GMFCS III
6	Male	5	26.0	110	SMA	Type II

**Table 3:** Vital signs before and after device use. SMA: Spinal Muscular Atrophy; CP: Cerebral Palsy; Dx: diagnostic; BP: blood pressure; % O_2_: oxygen saturation.

Dx	Heart Rate		Respiratory Rate		Systolic BP		Dyastolic BP		% O_2_	
	Before	After	Before	After	Before	After	Before	After	Before	After
SMA	105.7 ± 6.6	103.9 ± 4.4	21.7 ± 3.3	23.3 ± 3.2	96.4 ± 6.9	101.0 ± 6.4	61.0 ± 3.6	62.0 ± 9.1	97.6 ± 0.5	97.7 ± 0.7
SMA	106.7 ± 6.4	99.2 ± 6.6	22.2 ± 3.0	20.9 ± 3.0	101.8 ± 6.2	96.8 ± 3.4	69.0 ± 5.0	60.7 ± 4.1	98.3 ± 0.9	97.7 ± 0.5
CP	96.2 ± 5.7	103.0 ± 6.7	20.4 ± 2.6	19.4 ± 3.0	93.2 ± 7.4	83.1 ± 2.2	54.3 ± 5.0	58.9 ± 4.1	97.4 ± 0.7	97.7 ± 1.0
CP	104.7 ± 9.2	102.1 ± 7.1	22.9 ± 2.8	19.9 ± 3.0	94.0 ± 4.2	93.7 ± 5.9	60.1 ± 7.7	58.1 ± 6.3	97.5 ± 0.7	97.1 ± 0.9
CP	101.9 ± 6.8	106.+ ± 7.2	25.0 ± 3.2	26.0 ± 1.4	85.3 ± 6.1	88.6 ± 7.1	50.9 ± 4.3	50.9 ± 6.8	98.0 ± 0.5	97.8 ± 0.8
SMA	110.4 ± 5.3	105.7 ± 4.4	21.1 ± 2.8	22.3 ± 2.3	101.1 ± 4.4	98.4 ± 5.6	67.8 ± 7.2	65.2 ± 5.9	97.8 ± 0.4	97.6 ± 0.5

**Table 4:** Score per item and children’s responses to the QUEST 2.1. CP: Cerebral Palsy; SMA: Spinal Muscular Atrophy. Note: punctuations from 1 (delighted) to 7 (terrible).

Participant		1	2	3	4	5	6
DEVICE	Size	1	3	1	1	1	1
	Weight	3	3	4	2	5	1
	How easy it is to move	4	5	4	1	4	1
	How it looks	2	1	3	1	1	2
	How easy is to use	3	4	1	1	5	1
	Time it takes to set up	4	3	1	1	7	1
	Reliability	1	1	4	2	1	1
	Meets your needs	4	1	5	4	6	1
SERVICES	Advice given on what technology is best	1	1	2	1	1	1
	Time it took to get	6	7	2	2	6	6
	Help given if it is not working properly	1	4	1	1	1	1
	Advice given on how to use it	1	1	1	1	1	1
Median (IQR) score per participant		2.5 (3.0)	3.0 (3.0)	2.0 (3.0)	1.0 (1.0)	2.5 (4.7)	1.0 (0.0)

**Table 5:** Score per item and therapist in the QUEST 2.0. T: therapist. Note: punctuations range from 1 (not satisfied at all) to 5 (very satisfied).

	How Satisfied Are You with…	T1	T2
DEVICE	the dimensions (size, height, length, width)?	5	5
	the weight of your assistive device?	4	5
	the ease in adjusting (fixing, fastening) the parts?	5	4
	how safe and secure your assistive device is?	4	4
	the durability (endurance, resistance to wear)?	4	4
	how easy it is to use your assistive device?	5	5
	how comfortable your assistive device is?	5	4
	how effective your assistive device is?	5	4
SERVICES	the service delivery program in which you obtained your assistive device?	5	5
	the repairs and servicing provided?	5	5
	the quality of the professional services you received?	5	5
	the follow-up services (continuing support services)?	5	5
Median (IQR) score per therapist		5.0 (0.0)	5.0 (0.0)

## Data Availability

The data that support the findings of this study are available, but restrictions apply to the availability of these data, which were used under license for the current study, and so they are not publicly available. However, data are available from the authors upon reasonable request and with permission of the corresponding author.
